# Postoperative evaluation of interleukin-8 and C1q/TNF-related protein-12 in patients undergoing coronary artery bypass graft surgery

**DOI:** 10.1590/1806-9282.20240877

**Published:** 2025-01-10

**Authors:** Maryam Tahmasebi Sisakht, Hossein Pourghadamyari, Tania Dehesh, Zohreh Ramezani Karim, Mahdieh Nazari-Robati

**Affiliations:** 1 Physiology Research Center, Institute of Neuropharmacology, Kerman University of Medical Sciences – Kerman, Iran.; 2 Applied Cellular and Molecular Research Center, Kerman University of Medical Sciences – Kerman, Iran.; 3 Department of Clinical Biochemistry, Faculty of Medicine, Kerman University of Medical Sciences – Kerman, Iran.; 4 Department of Biostatistics and Epidemiology, School of Public Health, Kerman University of Medical Sciences – Kerman, Iran.

**Keywords:** Coronary artery bypass grafting, Interleukin-8, C1q/TNF-related protein-12

## Abstract

**OBJECTIVE:**

Coronary artery bypass graft surgery is one of the most frequently performed surgeries worldwide. Coronary artery bypass graft surgery induces an inflammatory response. Interleukin-8 is a pro-inflammatory cytokine that plays a role in the pathogenesis of cardiovascular diseases. C1q/TNF-related protein-12 is implicated in mitigating inflammation and cardiomyocyte damage. This study aimed to compare interleukin-8 and C1q/TNF-related protein-12 levels before and 45 days after coronary artery bypass graft surgery.

**METHODS:**

A total of 43 patients who underwent coronary artery bypass graft surgery were studied. Serum concentrations of interleukin-8 and C1q/TNF-related protein-12 were evaluated using the enzyme-linked immunosorbent assay method before and 45 days after the surgery.

**RESULTS:**

No significant differences were observed in interleukin-8 levels between pre- and post-coronary artery bypass graft surgery (p=0.077). However, serum levels of C1q/TNF-related protein-12 were found to be lower 45 days after coronary artery bypass graft surgery compared to pre-surgery levels (p<0.001). Moreover, changes in C1q/TNF-related protein-12 were not associated with diabetes, hypertension, and body mass index (p>0.05), but C1q/TNF-related protein-12 alterations were found to be associated with opium addiction.

**CONCLUSION:**

These findings suggest that the evaluation of C1q/TNF-related protein-12 can be beneficial for the late assessment of post-coronary artery bypass graft surgery inflammation. The reduction of C1q/TNF-related protein-12 levels might indicate increased levels of inflammation after surgery at this time point, which requires the assessment of further inflammatory factors to confirm this finding.

## INTRODUCTION

Coronary artery disease (CAD) is a complex and chronic condition, and a variety of factors, including inflammatory response, metabolic dysregulation, and endothelial dysfunction, are involved in its onset and progression. Despite advancements in preventive and therapeutic interventions, CAD remains the primary cause of death around the world^1^. CAD commonly requires invasive treatment such as percutaneous coronary intervention (PCI) or coronary artery bypass graft surgery (CABG). CABG has been shown to often trigger a systemic inflammatory response that contributes to postoperative complications^2^. The inflammatory response in patients undergoing CABG is induced through complex interactions with multiple pathways, resulting in the generation of cytokines and other inflammatory mediators^3^.

Interleukin (IL)-8 is a pro-inflammatory cytokine generated by various cell types^4^. As a potent chemotactic agent, IL-8 recruits neutrophils to the injured myocardium, causing inflammation^5^. In addition, previous research has indicated that IL-8 plays a role in the development and progression of atherosclerosis and its associated complications^6^. In the field of interventional cardiology, IL-8 levels have been shown to rise after procedures such as PCI or CABG, which is linked to the progression of tissue damage and the course of the disease. Hence, measuring the levels of this cytokine can aid in the diagnosis of high-risk patients^7^.

The adipokine C1q/TNF-related protein-12 (CTRP12) is a member of the CTRP family that is highly expressed in adipocytes. CTRP12 plays a significant role in insulin sensitivity and reducing inflammation^8^. Additionally, it exerts a protective effect against the development of atherosclerosis by inhibiting inflammatory responses^9^. A recent study found that CTRP12 ameliorates cardiomyocyte damage induced by lipopolysaccharides through lowering the levels of inflammation and apoptosis^10^. Furthermore, a decline in CTRP12 levels has been reported in patients with CAD^11^. Inflammatory responses are expected to be activated during or after CABG. Understanding the changes in inflammatory indicators can help to improve postoperative patient care. However, few investigations have examined late postoperative inflammatory responses. Therefore, in this study, the levels of IL-8 and CTRP12 were investigated before and 45 days after CABG.

## METHODS

### Study approval and patient characteristics

This study was conducted in accordance with the Declaration of Helsinki and approved by the Ethics Committee of Kerman University of Medical Sciences (IR.KMU.REC.1402.307). All patients gave informed consent to participate in this study. A total of 43 patients were admitted to Shafa Hospital (Kerman, Iran) to have an elective on-pump CABG. Inclusion criteria were age younger than 80 years and preoperative ejection fraction greater than 30%. Exclusion criteria were liver or renal diseases, cancer, acute myocardial infarction, infectious diseases, and medications with immune-modulating agents such as steroids before surgery^12^. Information on age, addiction, diabetes, and medications was obtained through a questionnaire. The body mass index (BMI) was calculated using the standard formula [weight (kg)/height (m^2^)]. Blood pressure was also measured in a seated position after 15 min of rest.

### Blood sampling

Blood samples were taken after 10 h fasting at two time points. The first sample was collected on the day before surgery, and the second sample was taken 45 days after surgical intervention when patients underwent a follow-up visit.

### Assay of interleukin-8 and C1q/TNF-related protein-12

The levels of IL-8 and CTRP12 were measured in serum using enzyme-linked immunosorbent assay (ELISA) kits according to the manufacturer’s instructions (ZellBio, Germany).

### Statistical analysis

All statistical analyses were conducted using the Statistical Package for the Social Sciences (SPSS) 26 software (SPSS Inc., IL, USA). Continuous variables were expressed as mean±SD. Categorical data were presented as absolute numbers and percentages. The normality of data distribution was assessed by the Shapiro-Wilk test. Changes in IL-8 and CTRP12 at two time points were analyzed using the Wilcoxon test. Longitudinal changes in IL-8 and CTRP12 were also evaluated by a generalized estimating equation (GEE) model with adjustment for confounding variables. Spearman rank correlation was used to investigate the correlation between IL-8 and CTRP12 levels. A p<0.05 was considered statistically significant.

## RESULTS

In this study, we examined a cohort of 43 participants, including 25 men and 18 women. The mean age of the participants was 61.16±6.35 years, and the average BMI was 26.25±2.65 kg/m^2^. When it came to health conditions, 46.5% of the participants had diabetes, and 41.9% had hypertension. Additionally, 18.6% of the participants reported having an addiction issue (Table 1).

**Table 1:** Demographic and clinical characteristics of patients.

Variable	Value
Age (years), mean±SD	61.16±6.35
**Gender**	
Men, n (%)	25 (58.1)
Women, n (%)	18 (41.9)
BMI (kg/m^2^), mean±SD	26.25±2.65
Diabetes, n (%)	20 (46.5)
Hypertension, n (%)	18 (41.9)
Addiction, n (%)	8 (18.6)

BMI: body mass index; SD: standard deviation.

Table 2 shows the mean values of IL-8 and CTRP12 at baseline and 45 days after CABG. The results did not reveal any statistically significant changes in the levels of IL-8 between these time points (p=0.068). However, a significant decrease was observed in CTRP12 levels from baseline to day 45 post-CABG (p<0.001). In addition, the difference in IL-8 levels was not significant (p=0.077) after adjustment of potential confounders using the GEE model, whereas the reduction of CTRP12 remained significant in this model (p<0.001). Moreover, our data did not show any statistically significant effects of BMI, diabetes, hypertension, and addiction in relation to IL8 (p>0.05). Similarly, BMI, diabetes, and hypertension did not have any significant effects on CTRP12 (p>0.05), but addiction was positively associated with a reduction in CTRP12 levels (p=0.01). According to the data, the patients who did not have addiction showed a significantly higher reduction in CTRP12 compared to addicted patients (Table 3). Our results did not also indicate any significant correlations between IL-8 and CTRP12 before and after surgery (r=0.129, p=0.411; r=0.281, p=0.155).

**Table 2:** Comparison of the serum levels of interleukin-8 and C1q/TNF-related protein-12 before and 45 days after coronary artery bypass graft surgery.

Variable	Pre-CABG	Post-CABG	p-value
IL-8 (pg/mL)	243.62±32.88	236.02±30.53	0.068
CTRP12 (pg/mL)	354.42±66.98	290.02±59.57	<0.001

CABG: coronary artery bypass grafting; IL-8: interleukin-8; CTRP12: C1q/TNF-related protein-12. Data are presented as mean±SD.

**Table 3:** Generalized estimating equation for investigating interleukin-8 and C1q/TNF-related protein-12 variations during time.

	β	95%CI for β	p-value
**IL-8**			
Time (post- vs. pre-CABG)	-7.60	-9.12, -2.24	0.077
Age	-0.66	-1.97, 0.65	0.325
Gender (women vs. men)	13.22	-7.63, 24.08	0.214
BMI	-0.43	-2.51, 1.64	0.681
Diabetes (no vs. yes)	2.69	-1.71, 7.08	0.762
Hypertension (no vs. yes)	-1.37	-4.64, 2.91	0.861
Addiction (no vs. yes)	-16.78	-18.46, -1.22	0.147
**CTRP12**			
Time (post vs. pre-CABG)	-64.39	-71.21, -42.24	<0.001
Age	0.87	-1.77, 2.51	0.518
Gender (women vs. men)	18.48	-6.24, 20.27	0.080
BMI	0.61	-3.22, 2.51	0.802
Diabetes (no vs. yes)	5.61	-2.91, 7.39	0.735
Hypertension (no vs. yes)	12.27	-3.62, 16.41	0.407
Addiction (no vs. yes)	-61.91	-71.24, -42.72	0.010

β: longitudinal (GEE) regression coefficient; CI: confidence intervals; CABG: coronary artery bypass grafting; BMI: body mass index; IL-8: interleukin-8; CTRP12: C1q/TNF-related protein-12.

## DISCUSSION

Despite advancements in the therapeutic approaches for CAD, CABG remains one of the most frequently performed surgical procedures worldwide. CABG activates various molecular pathways that lead to an elevated inflammatory response, which contributes to the occurrence of numerous post-CABG complications and myocardial damage^13^. The investigation of certain inflammatory markers has revealed that their levels did not return to the baseline even after 30 days post-CABG^14^. Therefore, late assessment of inflammatory markers can be beneficial in mitigating the late occurrence of several CABG-related complications. In the present study, IL-8 and CTRP12 levels were evaluated before and 45 days after CABG, and the results revealed that the serum level of IL-8 did not change significantly post-surgery compared to pre-surgery levels, while the CTRP12 levels decreased significantly.

IL-8 is a potent chemotactic agent primarily produced by monocytes. The association between elevated IL-8 levels and the development of cardiovascular disorders has been reported in several studies^5^,^15^. Furthermore, an increase in IL-8 levels has been observed after CABG, with the myocardium and lungs serving as the main sources of its generation during cardiopulmonary bypass^16^. Our findings did not show a statistically significant difference in IL-8 levels 45 days after CABG compared to the baseline. This difference was also not significant after adjustment for the effects of variables including diabetes, hypertension, addiction, and BMI. Diegeler et al. showed that IL-8 levels increase during the initial period after CABG. In their study, the maximum level of IL-8 was reported 48 h post-surgery, remaining higher than the baseline for up to 6 days after CABG^17^. Another study demonstrated a peak in the level of IL-8 2 days after CABG, which remained relatively constant until 5 days after the surgery^18^. In the study by Mirhafez et al., an increase in IL-8 levels was also reported 24 h after on-pump CABG^19^. Although the time points reported in these studies are different, the results generally show that IL-8 has the earliest and highest peak levels between the later stages of CABG and very early hours after surgery, and its levels gradually decrease thereafter. Probably for this reason, the levels of IL-8 on day 45 post-CABG were not statistically different from the baseline levels.

CTRP12 is a member of the CTRP family, which is abundantly produced by adipose tissue^20^. Several studies have demonstrated the protective effects of CTRP12 against various cardiometabolic disorders^9^,^21^. The results of this study revealed that CTRP12 levels decreased significantly 45 days after CABG. After adjusting for the effect of diabetes, BMI, hypertension, and addiction, it was found that the change in the CTRP12 level was associated with addiction to opium. To the best of our knowledge, this is the first study to investigate CTRP12 levels after CABG. Decreased levels of CTRP12 have been reported in other cardiovascular diseases. Fadaei et al. found that CTRP12 levels were diminished in CAD and were associated with the risk of developing this disease^11^. Decreased levels of CTRP12 in patients with acute myocardial infarction were reported in the study by Babapour et al. Moreover, similar to our study, they found no association between the serum levels of CTRP12 and BMI or blood pressure^22^. Reduced expression of CTRP12 has been reported in ischemia-reperfusion-induced myocardial tissues and hypoxia-regeneration-induced cardiomyocytes. Conversely, an elevated expression of CTRP12 has protective effects and minimizes myocardial cell damage, inflammation, and apoptosis^23^. The inverse relationship between CTRP12 levels and the levels of tumor necrosis factor-alpha (TNFα) and IL-6 has also been confirmed in CAD patients^11^.

According to the results of these studies, CTRP12 has protective effects against inflammatory responses, and the observed decrease in its levels in patients 45 days post-CABG surgery may be associated with increased levels of inflammation during this time. On the contrary, IL-8 levels did not change compared to pre-surgery levels. Additionally, our results did not show a correlation between IL-8 and CTRP12 levels before and after CABG, which may be due to the small sample size. Therefore, a study on a larger number of patients undergoing CABG is required. Additionally, in this study, it was found that opium addiction did not impact the levels of IL-8, while it was associated with a reduction in CTRP12 levels. There are conflicting reports regarding the effect of opium on the immune system’s response to various stresses. While numerous studies suggest that opium use can increase the production of inflammatory cytokines^24^, other studies have reported a decrease in the level of inflammatory factors among opium users^25^. However, no study has investigated the effect of opium on CTRP12 levels.

This study has several limitations. First, the study population size was small. Hence, these findings should be confirmed in a larger sample size in subsequent studies. In addition, only IL-8 was assessed in this study. In order to reach the correct conclusion regarding the state of post-CABG inflammation, it is necessary to examine other pivotal cytokines, such as TNFα, IL-1β, and IL-6. Another limitation was that IL-8 and CTRP12 were measured at one time point after CABG. Finally, other intervening factors, such as medications and dietary patterns, were not examined.

## CONCLUSION

Overall, the data suggest that CABG surgery was associated with decreased CTRP12 levels 45 days after the surgery. A reduction in CTRP12 levels may thus lower its protective effects against inflammation. However, no significant change was observed in the levels of the pro-inflammatory cytokine IL-8 at this time point. Therefore, evaluating other inflammatory factors is recommended to elucidate the relationship between post-CABG inflammatory responses and the reduction of CTRP12.
